# Recommendations on recording harms in randomised controlled trials of behaviour change interventions

**DOI:** 10.1136/bmj-2023-077418

**Published:** 2024-10-02

**Authors:** Diana Papaioannou, Sienna Hamer-Kiwacz, Cara Mooney, Kirsty Sprange, Cindy Cooper, Alicia O’Cathain

**Affiliations:** 1 Clinical Trials Research Unit, School of Health and Related Research, University of Sheffield, Sheffield, S1 4DA, UK; 2 Nottingham Clinical Trials Unit, University of Nottingham, Nottingham, UK; 3 Health and Care Research Unit, School of Health and Related Research, University of Sheffield, Sheffield, UK

## Abstract

Harms are possible from behaviour change interventions, such as the worsening of a health behaviour intended for change (rebound effect), improving a health behaviour but with subsequent worsening of another behaviour (risk compensation), and participants feeling targeted or stigmatised by an intervention. The processes and definitions originally designed to record harms within drug trials are typically followed to record harms in trials of behaviour change interventions owing to the lack of alternative guidance. Therefore, important harms could be missed in the evaluations of behaviour change interventions or irrelevant harms data may be recorded, leading to inefficiency. This paper presents evidence informed recommendations on how to record harms in randomised controlled trials of behaviour change interventions.

Randomised controlled trials evaluate the risks and harms, as well as the benefits, of interventions. However, in randomised controlled trials of behaviour change interventions, such as psychological therapies, public health, or lifestyle interventions, the recording of harms is poor and inconsistent.^1^ ^2^ ^3^ ^4^

There might be a misconception that harms are not possible from behaviour change interventions,^1^ despite evidence to the contrary.^2^ ^3^ ^5^ ^6^ Trialists typically use the Good Clinical Practice definitions^7^ where harms are referred to as “adverse events” and defined as “untoward medical occurrences.” Serious adverse events are those that result in death, life threatening episodes, or admission to hospital, for example. Trialists use these definitions in randomised controlled trials of behaviour change interventions because they are the definitions required by the Health Research Authority, the approving body for research in the NHS in England.^8^ These definitions are also familiar to trialists and used within the procedures and processes (standard operating procedures) of Clinical Trials Units across the UK.

There is a need to consider harm more widely in the context of randomised controlled trials of behaviour change interventions. Use of the definitions of harms originally devised for drug trials^7^ risks missing important harms relevant to behaviour change interventions^1^ and could be an inefficient process because staff and participants can spend a disproportionate amount of time recording irrelevant events.^9^ ^10^

We have developed evidence informed recommendations to improve the recording of harms arising in randomised controlled trials of behaviour change interventions. We aimed to improve the efficiency of recording harms and ensure that recording harms better reflects the harms relevant to behaviour change interventions.

**Summary points**

There could be a misconception that harms cannot be caused by behaviour change interventions
Reliance on processes and definitions originally designed to record harms in drug trials might contribute to poor, inconsistent, and inefficient recording of harms in randomised controlled trials of behaviour change interventions
These new, evidence informed, recommendations provide guidance on how to identify plausible harms from a behaviour change intervention; what harms might be recorded as part of a proportionate approach; how to collect data on harms (eg, using both direct and open ended questions and qualitative research); and multidisciplinary team input with a variety of perspectives, in particular from patient and public involvement representatives
These recommendations were developed using a systematic scoping review, qualitative interviews, and online workshops with experts involved in the design and implementation of randomised controlled trials

## Development of recommendations

The recommendations are based on three sources—firstly, from a systematic scoping review of categories, definitions, or mechanisms of harms from behaviour change interventions, methods of identifying plausible harms, and general recommendations for recording harms.^11^ We also undertook 15 qualitative interviews and organised three focus groups with 29 experts in trial design and implementation. These experts included trial managers, chief investigators, clinical trials unit directors, statisticians, and patient and public representatives.^12^ Thirdly, we triangulated findings from the systematic scoping review and the qualitative interview study to draft recommendations that were then reviewed in two online workshops by 14 multidisciplinary experts in randomised controlled trials. Appendix 1 in the supplementary material presents more information about these workshops.

One week before the workshops, participants were sent the draft recommendations along with a form for feedback. During the workshops, the findings of the scoping review and qualitative study were presented, and four topics were discussed: identifying and deciding what harms to record, collecting harms, summarised guidance, and implementation and dissemination. Refinements were made to the recommendations based on these workshops. A project advisory group provided oversight throughout the project and approved the final version of the recommendations.

The original intention had been to undertake a two stage, online eDelphi study and consensus meeting instead of the online workshops.^13^ However, the qualitative study findings indicated that this area is complex, where consensus statements would not be achievable without extensive explanation alongside each statement, resulting in a Delphi study that would be difficult to interpret. A Delphi study was therefore not attempted.

## The recommendations

We propose these recommendations as a set of guiding principles in an area where there is no guidance. The overarching aim is to stimulate research teams into considering that harm is possible from these types of interventions, that harms might not be captured by definitions that they usually use, and how harms can be recorded efficiently.

**Fig 1:**
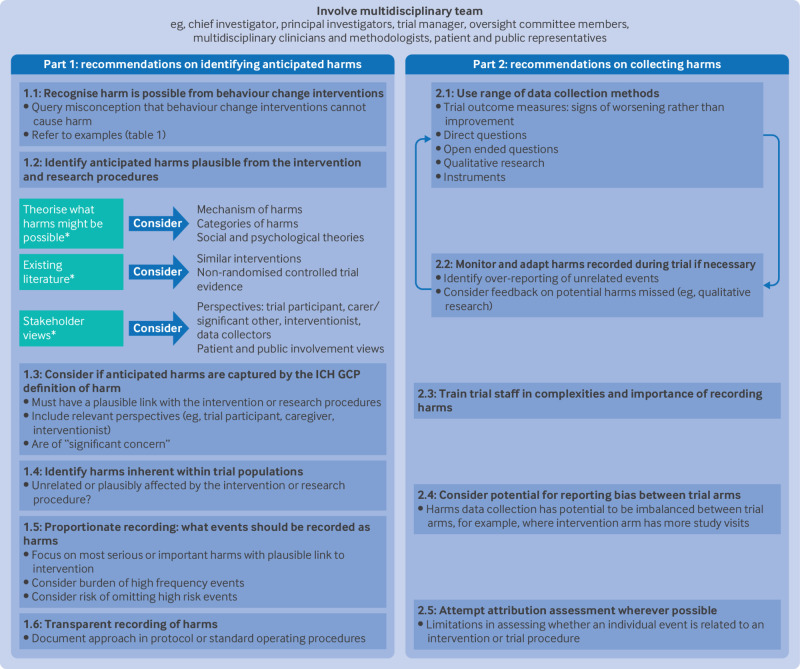
Overview of the recommendations for recording harms in behaviour change intervention trials. ICH GCP=International Council For Harmonisation of Technical Requirements for Pharmaceuticals for Human Use Guideline for Good Clinical Practice. *Steps based on dark logic model approach from Bonell et al^2^

Figure 1 summarises the recommendations. We include the participation of a multidisciplinary trial team as a cross cutting principle that is central to all recommendations on recording harms in randomised controlled trials of behaviour change interventions, rather than as a discrete stage. Figure 1 positions this principle as applicable to all recommendations. The recommendations are then divided into two parts. Part 1 describes the guiding principles to identify anticipated harms*.* Part 2 describes collecting harms, including unanticipated harms. Throughout this article, we use a hypothetical worked example to illustrate the application of the recommendations. The recommendations are also summarised as a checklist (supplementary information).

## Involve the multidisciplinary trial team (cross cutting principle)

An underlying assumption that underpins the recommendations is involvement of the multidisciplinary trial team. We recommend discussing the approach to recording harms early on within the trial team. Trials are run by individuals with different expertise (eg, clinical, trial methodology, or lived experience), which offers different perspectives and input on potential harms from behaviour change interventions. A strong theme from the qualitative study was the importance of seeking a range of views and opinions when considering harms, to avoid the approach of recording harms being determined by one or two individuals. The involvement of patient and public representatives is essential, particularly when identifying the harms that might be plausible from an intervention and for views on acceptability of what ought to be recorded as a harm.

The scoping review emphasised the importance of recording harms being a shared responsibility. Independent oversight committees, such as the trial steering committee or data monitoring committee who oversee the conduct and safety of randomised controlled trials, are also essential to engage from the outset. These committees can review and ratify the approach to harms recording.

## Part 1: Identifying anticipated harms

Part 1 describes six recommendations for identifying the harms anticipated from a behaviour change intervention.

### 1.1: Recognise harm is possible from behaviour change interventions

There could be a misconception that harms from behaviour change interventions are not possible; a review of trial protocols identified that 10% did not record harms because “the intervention was behavioural and not expected to cause harm.”^1^ In the context of the Good Clinical Practice definitions of serious harm (eg, events resulting in death, life threatening episode, or admission to hospital),^7^ behaviour change interventions might be low risk. However, other important harms might need to be considered to fully examine the risks and benefits of the intervention. Evidence from our scoping review suggests that these types of interventions can cause harm.^11^ Most participants in our qualitative study were not aware of the literature identified in the scoping review. Therefore, discussion of examples of harm might be a first step in ensuring that the trial team understands that harm is possible from behaviour change interventions. Table 1 presents three examples of harm within different behaviour change interventions; further examples are available in our scoping review.^11^

**Table 1:** Empirical examples of harms within behaviour change interventions*

Section	Example 1	Example 2	Example 3
Population	Inflammatory bowel disease†^14^	Children aged 12-14 years‡ who had taken part in a social and emotional learning intervention^15^	Adult meditation participants^16^
Intervention	Social support or peer support that could include various sources, such as family, friends, coworkers, and face-to-face or online support groups	Targeted social and emotional learning intervention that aims to develop social and emotional competencies in children and young people, including a targeted student support group. Two trained facilitators with a group of 8-12 students delivered eight sessions weekly	Meditation or meditation based therapeutic interventions, including mindfulness
Study design	Qualitative interview study	Qualitative interview study	Systematic review that included experimental and observational studies
Harms	Social support sometimes resulted in distressing conversations for patients, which led to: • Confrontation with unwanted information by social contacts • Undesirable reactions from others (pity, over-reacting, unwanted attention) • Some patients feeling more anxious about their current and future health, by increasing uncertainty and pessimism (eg, confrontation with a possible negative future or interaction with people who were feeling much better than the patient) • Some patients feeling weak, not in control, helpless, and dominated by their illness.	Four harmful processes were identified: • Negative labelling: identification to take part in the intervention caused further exclusion and stigma for children; participants thought the teachers “hated them more” for choosing them to be in the group • Coveted labelling: identification to take part in the intervention sometimes improved children’s position within their peer groups, which could reduce their engagement with the intervention and cause further anti-school attitudes and behaviours • Seeking safety: targeting of pre-existing friendship groups caused students to stay within these groups and create outsiders who were not part of the group, causing isolation to these individuals • Students might brag to friendship groups about anti-social and even illegal activities they had undertaken, therefore reinforcing them, and encouraging others to do similar, potentially worse behaviour.	Of 83 studies analysed, 55 (66%) reported at least one adverse event. Total prevalence of adverse events (8.3%) varied across study designs (3.7% for experimental studies, 33.2% for observational studies) • Three categories of adverse events were identified: psychiatric (49% studies), somatic (31%), neurological/cognitive (20%) • Most common adverse events were psychiatric: anxiety (18 studies), depression (15), psychotic or delusional symptoms (10), dissociation or depersonalisation (9), and fear or terror (9) • Most common somatic adverse events were stress or physical tension (11 studies), pain (9) and gastrointestinal problems (6) • Neurological or cognitive adverse events included cognitive anomalous experiences, thought disorganisation, amnesia, perceptual hypersensitivity, and impaired memory reliability • Longer term effects of more than six months were reported in nine (17%) studies.

* Further examples available in the systematic scoping review.^11^

† Includes Crohn’s disease, ulcerative colitis, and indeterminate colitis.

‡ From four mixed sex and socioeconomically and academically diverse secondary schools in south Wales, UK.

### 1.2: Identify anticipated harms from an intervention: theorise, search the literature, and involve stakeholders

The Health Research Authority expect trial protocols to list harms expected from an intervention and within a population.^8^ However, a systematic review identified that only 15% of trial protocols did so in behaviour change trials.^1^ For drug trials, expected harms are contained in the reference safety information, such as the summary of product characteristics or investigator’s brochure. No such documents exist in non-drug trials, and therefore trialists might be uncertain in how to identify expected (which we refer to as anticipated) harms in behaviour change interventions. Trial teams might also need to consider if research procedures (eg, questionnaires) might cause harm.

Harms anticipated from an intervention can be best identified by following a three step process described by Bonell et al,^2^ known as the dark logic model. We provide a worked example in box 1. The three steps are:

Box 1 Identifying anticipated harms from a behaviour change intervention (worked example)*

**Behaviour change trial (hypothetical)**

Population: Risky drinkers (ie, drinkers prone to alcohol misuse) aged >65 years, identified by their general practitioner.
Intervention: One month’s abstinence from drinking, plus one workshop led by general practitioners.
Comparison: Reducing drinking following tips from the NHS website.
Outcome: Change in self-reported weekly alcohol consumption at six months, via the alcohol use disorders identification test (AUDIT) self-report measure.

**Step 1: Theorise**

Three mechanisms and plausible† harms are identified by the trial management group:

- Rebound or boomerang effects: risky drinking worsens
- Risk compensation: replacing risky drinking with another behaviour (could be positive or negative)
- Drinker has feelings of failure or lack of self-efficacy if unable to abstain from alcohol, leading to shame, stigma, guilt; in turn could contribute to rebound effect.

**Step 2: Search the literature**

Literature identifies alcohol withdrawal symptoms as potential harms, but information is limited on other unintended consequences, although some commentary on the importance of recording unintended consequences.^17^ ^18^

**Step 3: Include stakeholder input**

Patient and public involvement representatives and other stakeholders (eg, general practitioners) are presented the theoretical mechanisms by which harm occur and agree that they are important. Stakeholders (including general practitioners) are particularly concerned with feelings of failure or guilt leading to a rebound effect. They say that a temporary change (within the month of alcohol abstinence) in other health behaviours (risk compensation) is not of concern†, but are concerned about negative health behaviour changes in the longer term that are beyond the period of alcohol abstinence (eg, eating more unhealthy food, online game use).

**Perspectives†**

Relevant individuals who might identify harm:

- Trial participants
- General practitioners

Significant others and family members are noted as other relevant persons, but might not have the opportunity to collect data from these individuals.

**Serious or important harms†**

Withdrawal symptoms for alcohol dependency are well known, although study inclusion criteria to exclude those individuals with alcohol dependency should limit these symptoms. These withdrawal symptoms will be picked up by the standard Good Clinical Practice definition of harm (ie, adverse event^7^).

Excessive and highly abnormal risk compensation behaviours might be captured as adverse events; however, they would not qualify as a serious adverse event.^7^ These events are important to capture in real time, thus these harms are defined as serious harms. The trial management group should adjudicate what is excessive or abnormal on a case-by-case basis.

*Box B in the supplementary material includes another worked example.†Key concept in defining harm (see section 1.3 below).

1. Theorise: Expertise might exist within the trial team to apply social and psychological theories to consider how harms might occur.^2^ The systematic scoping review^11^ identified categories and mechanisms of harms (box A in supplementary material). We recommend that trial teams consider whether these categories and mechanisms apply to their intervention. For example, if an intervention is group based, a mechanism for harm could lead to unhelpful knowledge exchange among trial participants, which might promote undesirable behaviours.
2. Search the literature: The literature is unlikely to cover the exact intervention being evaluated in the trial. Use a broad literature search by considering similar interventions. Owing to the paucity of evidence of harms within behaviour change interventions in randomised controlled trials, trial teams might need to consider other study designs. Search terms will need to reflect how authors might describe harm (eg, “unintended”/“unanticipated” or “unwanted”/“unplanned”; “event”/“effect”(s), “harm”/“consequence”(s), or “impact”/“repercussion”(s)).
3. Include stakeholder input: Stakeholder perspectives are important for identifying potential harms and determining the importance of those harms. These stakeholders might include patient and public representatives or those delivering the intervention (such as schoolteachers). Sharing examples of harms from previous behaviour change interventions might be helpful, even if not related to the intervention being evaluated, to demonstrate the types of harms possible and provide context.

### 1.3: Consider if anticipated harms are captured by the Good Clinical Practice definition of harm

Our qualitative study identified the current approach to recording harms in behaviour change intervention trials is driven by regulatory requirements and Clinical Trials Unit Standard operating procedures. This process involves defining harms as adverse events. An adverse event is any untoward medical occurrence in a patient or clinical investigation participant given a pharmaceutical product and that does not necessarily have to have a causal association with this treatment. Each adverse event is assessed for seriousness, expectedness, and relatedness to the intervention. A serious adverse event is an adverse event that at any dose results in death or is life threatening (ie, requires hospital admission or a prolongation of existing hospital stays, results in persistent or clinically significant disability or incapacity, or is a congenital abnormality or birth defect). This assessment identifies serious adverse reactions and suspected unexpected serious adverse reactions, with the second group of events being reportable to regulatory authorities according to strict timelines.

The main problems with the current approach are that it could miss relevant and important harms that are not medical events or consequences, the medical terminology might not be appropriate for all harms that arise from behaviour change interventions,^12^ and it might be highly inefficient owing to excessive time spent recording large numbers of irrelevant events.^9^ ^10^ ^12^

Once trial teams have identified anticipated harms from a behaviour change intervention (see section 1.2), we recommend that trial teams consider whether these harms are captured by the Good Clinical Practice definitions.^7^ Harm might not always be a medical event or consequence, as demonstrated by the examples in table 1. Furthermore, the  Consolidated Standards of Reporting Trials (CONSORT) harms extension defines harm as “the totality of possible adverse consequences of an intervention or therapy; they are the direct opposite of benefits, against which they must be compared,”^19^ and recommends against using terms such as “adverse events” and “side effects.”

Researchers might be uncertain as to what constitutes an adverse consequence, according to the CONSORT harms extension definition.^19^ We identified three key aspects that might help define harm arising from a behaviour change intervention.^11^ ^12^ Firstly, the harm must be plausibly caused by the intervention. Secondly, perspectives beyond just the participant might be important to consider, such as family members or trial personnel. Thirdly, owing to potential subjectivity on what may be viewed as harmful, an event must be “of concern” to the participant or other relevant person for it to be considered harmful. Subjectivity is inherent in harm perception, because individuals might view the same event differently, and vary in their response (ie, some individuals might think of an event as a minor discomfort, whereas others might be more concerned). Therefore, defining what is “of concern” is difficult; participants in our qualitative study found this process to be complex. Involving patient and public representatives could provide insight into perceived importance of harms.

We propose the following definition of harm within a behaviour change intervention trial: “An event or an unintended consequence plausibly caused by a trial intervention or research procedure, and which is of concern to a study participant or other relevant person.” Note that consent will be required if there is collection of harms from non-trial participants, for example, significant others or family members.

#### Serious or important harms

Trial teams might want to consider what constitutes a serious or important harm that requires real time notification, which might be harms that do not meet the standard Good Clinical Practice definitions for seriousness.^7^ This could include social harms, such as an adolescent running away in a family therapy trial, which are important to report promptly to the trial team. We apply the principles from section 1.3 to our hypothetical example in box 1, which are helpful to consider during the three step process in section 1.2 (identifying anticipated harms).

### 1.4: Identify the common harmful events within trial populations

Harmful events inherent in a trial population (eg, hospital admissions for medical events occurring in an elderly population) often pose difficulties for trial teams in terms of whether such events should be recorded as a harm within a trial. Initially, we recommend that the trial team identifies the common harmful events within their trial population. Deciding on whether these events are to be recorded as harms within a randomised controlled trial can be considered as part of section 1.5 (proportionate recording).

### 1.5: Proportionate recording of harms: consider prioritising the most plausible or important harms

At this point, trial teams will have identified a list of anticipated harms that might or might not be captured using the Good Clinical Practice definition of harms.^7^ Trial staff can spend a disproportionate amount of time recording irrelevant events.^9^ ^10^ ^12^ Instead, trial teams could prioritise the most plausible, serious, and important harms to conserve trial resources.

Determining what events to prioritise or exclude in recording of harms is likely to be on a trial-by-trial basis, with multidisciplinary team input including patient and public involvement representatives. A reasonable explanation should be given for how the harm could be caused by the intervention (ie, plausibility). Decisions on what information to record might also consider the exclusion of events that occur frequently in both trial arms with limited evidence of causation from the intervention. Furthermore, a balance should be struck between risk of omission of harms and the burden on the trial team. Where a plausible link cannot be ruled out, and if such events are expected and serious in nature, a more cautious and conservative approach might be required because the risk of omission is high. For example, hospital admission for children and adolescents might be recorded in a psychological therapy trial. The low frequency of such events will not be burdensome to the trial team.

Decisions to exclude events from harm recording should have approval from the oversight committee and sponsor. Events not recorded as harms might still need to be reported through other trial protocols, such as safeguarding. Box 2 provides a worked example.

Box 2 **Proportionate recording of harms: consider prioritising the most plausible or important harms (worked example)**

**Behaviour change trial (hypothetical)**

Population: Risky drinkers (ie, drinkers prone to alcohol misuse)aged >65 years, identified by their general practitioner.
Intervention: One month’s abstinence from drinking, plus one workshop led by general practitioners.
Comparison: Reducing drinking following tips from the NHS website.
Outcome: Change in self-reported weekly alcohol consumption at six months, via the alcohol use disorders identification test (AUDIT) self-report measure.

**Harms included**

- All long term risk compensation and rebound effects to be recorded
- Feelings of failure, shame, and stigma to be recorded
- Collect for harms after the intervention and at six month follow-up to determine persistence of potential harms
- General practitioner’s or interventionist’s perspective of harm
- Serious harms: as defined in box 1.

**Harms excluded**

Risk compensation or rebound effects within the month of temporary abstinence, which are of less concern, are less important, and are excluded from recording.

### 1.6: Ensure transparency in recording harms: list anticipated harms in protocol as a minimum standard

The scoping review identified a need for better transparency in recording of harms. At a minimum, researchers should list plausible expected harms from the trial intervention or research procedures and harms expected in the population in the trial protocol. This is an expectation of the NHS Health Research Authority.^8^ We also recommend, as best practice, to document the methods used to identify harms plausibly caused by the intervention (eg, by the three step approach of the dark logic model^2^), and identify which harms are excluded from recording (with justification for doing so). This information could be included in the trial protocol or a standard operating procedure and can demonstrate that trialists have not cherry picked harms at the time of trial reporting.^20^ The sharing of harms data is also important. Rare and unpredictable harms are unlikely to be identified in one randomised controlled trial, but synthesising harms data across studies could enable identification of such events. However, the lack of consistency in harms reporting, even in the same health condition and same intervention, makes synthesising difficult.^21^

## Part 2: Collecting harms, including unanticipated harms

Part 2 provides five recommendations on how to collect harms. In our qualitative study, we found no best practice on recording harms in these types of trials. Processes for drug trials are typically followed in the absence of other guidance, but our interviewees recognised that this approach was not ideal.^12^ We have used the principles identified in our scoping review and interview study^11^ ^12^ and offered suggestions on how they might be put into use. Participants of the online workshop reviewed and refined these suggestions.

### 2.1: Use a range of data collection methods

The systematic scoping review identified that harms should be collected systematically by a range of data collection methods.^11^ ^21^ These methods include the trial outcome measures intended to measure efficacy, direct questions, open ended questions, qualitative research, and bespoke instruments to record harms,^22^ ^23^ ^24^ ^25^ ^26^ ^27^ ^28^ ^29^ ^30^ where they exist.

Trial outcome measures recording efficacy of the intervention offer an opportunity to capture harm. For example, where an intervention might plausibly worsen depression, a trial outcome measure such as a depression scale would detect this change. This approach relies on a data monitoring committee to view the accumulating data, although behaviour change intervention trials are frequently termed as low risk, and might not have such a committee. The literature has examples of interventions worsening the health behaviour intended for improvement.^2^ ^3^ Therefore, an independent oversight committee with access to the blinded data should be considered (sometimes a trial steering committee can perform this role).

Anticipated harms should be recorded systematically,^21^ by formulating a direct question or anticipated harms specified in the protocol. Naming specific events that might prime or influence a participant and introduce bias might raise concerns. However, behaviour change intervention trials routinely use patient reported outcome measures, a limitation that has the potential to introduce self-report bias.

Unanticipated harms should be considered because it is impossible to consider all potential harms that are plausible from an intervention at trial outset.^11^ ^31^ No approaches were found in the literature or qualitative study on how to do this. We suggest prompting participants with an open ended question (see example in box 3) or using qualitative research,^11^ ^12^ especially during the early stages of intervention evaluation (eg, feasibility studies or external pilots). Researchers should avoid any inadvertent unblinding of staff involved in reviewing open ended questions.

Box 3 **Use a range of data collection methods (worked example)**

**Behaviour change trial (hypothetical)**

Population: Risky drinkers (ie, drinkers prone to alcohol misuse) aged >65 years, identified by their general practitioner.
Intervention: One month’s abstinence from drinking, plus one workshop led by general practitioners.
Comparison: Reducing drinking following tips from the NHS website.
Outcome: Change in self-reported weekly alcohol consumption at six months, via the alcohol use disorders identification test (AUDIT) self-report measure.

**Trial outcome**

- Primary outcome is the AUDIT questionnaire, a self-report measure that assesses the nature and severity of alcohol misuse. This measure will pick up rebound effects (worsening of the health behaviour intended to change)
- The data monitoring committee charter notes the need to review accumulating data and check that there is no evidence for worsening of AUDIT score in the intervention group compared with the control group.

**Direct questions**

- While you were taking part in the study, did you notice a change in any of the behaviours below? For each behaviour, please indicate in the table whether this change concerned or worried you.

**Table:** 

Behaviour	Did you notice any change	If you noticed any of these changes, how much did this concern or worry you			
		Not concerned	A little concerned	Concerned	Very concerned
Increase in unhealthy eating	Yes/No				
Increase in smoking	Yes/No				
Increase in use of online gaming	Yes/No

- A questionnaire for the general practitioner or interventionist can also be used to incorporate questions on their perspective of harms.

**Open question and qualitative**

- Stakeholders and patient and public involvement representatives note the importance of collecting harms on fear, shame, or stigma but advise that this approach might be difficult as a direct question. Decide to capture this information by using open ended questions and qualitative interviews.
- Open ended question: When you took part in this study, was there anything that worried or concerned you? These could be events or feelings that you may or may not have expected from taking part in the research study or abstaining from alcohol.

### 2.2: Monitor and adapt the harms recorded during the trial if necessary

Flexibility is important,^11^ which might entail regular review of harms recorded during the trial to flag modifications required. For example, over-recording of events not plausibly caused by an intervention could occur. Conversely, trial teams might become aware that important harms are not being collected. Trial oversight committees can review and agree proposed adaptations to the recording of harms.

### 2.3: Train trial staff in the complexities and importance of recording harms

The trial team will need to understand the complexities and importance of recording harms.^11^ Examples of harms that fall outside the Good Clinical Practice definition might be useful (table 1; further examples available in our scoping review^11^). Where there is potential for data collectors to influence the reporting of harms data, for example, if sensitive harms data or potential for power imbalances exist (eg, in a trial of children and young people), the importance of an empathetic approach could be highlighted during training.

### 2.4: Consider the potential for reporting bias between trial arms

Considering the time points for data collection could reduce reporting bias.^11^ Harms data collection could be imbalanced between the trial arms, for example, where the intervention arm has more study visits. For remotely delivered interventions, opportunities to collect harms might also be limited.

### 2.5: Attempt attribution assessment wherever possible

There are limitations to assessing whether an individual event is related to an intervention or trial procedure. Researchers (who we interviewed) described difficulties in obtaining complete data on events to make attribution assessments, particularly where repeated requests for further information to assist with attribution assessment was not appropriate (eg, where an event was sensitive or distressing).^12^ Input from several trial team members might be required, which can be resource intensive. These difficulties do not have easy solutions, but we recommend where possible that attribution of events is attempted. Trial oversight committees (data monitoring or trial steering committees) can help assist with attribution assessment where an assessment cannot be made by the trial team. The difficulties in assessing whether an individual event or harm is related to the intervention underlines the importance of recording harms in both the intervention and control arms. In doing so, this will allow comparison on the rate of harms, as would be expected in any trial irrespective of intervention type.

## Summary

These new recommendations are offered as a set of guiding principles to help researchers identify and record harms arising in randomised controlled trials of behaviour change interventions. We hope that the recommendations will lead to more systematic, transparent, and accountable decision making in how to record harms within behaviour change intervention trials, while also paying attention to trial efficiency by proportionate recording of harms. However, we recognise this area is complex, and that trial teams will need to consider the recommendations make decisions on what harms to record and why on a case-by-case basis (ie, the decisions made might differ between randomised controlled trials). Multidisciplinary team input and trial oversight committee review is essential for this.

We encourage trial teams to publish worked examples of applying the recommendations. Work to improve recording of harms in specific interventions might also assist trial teams, for example, in the reporting of harms in digital interventions.^32^ ^33^ The inclusion of harms within core outcome sets could help in the systematic identification of harms in behaviour change trials, particularly focusing on those that are important to patients.^21^ Our key aim is to raise awareness that harms are possible from behaviour change interventions, and that trial teams give this topic consideration.

## Web extra

Extra material supplied by authors

Web appendix: Supplementary material

## Notes

Contributors: DP conceived the idea for the project and was the principal investigator. DP led the grant application for funding; CC, CM, AO’C, and KS provided input into the grant application. Work package 1 (reviews) contributors were DP, SH-K, and CM. Work package 2 (qualitative work) contributors were DP, SH-K, KS, and CM. Work package 3 (workshops) contributors were DP, SH-K, CM, AO’C, and CC. DP, SH-K, and CM contributed to all work packages, and led work package integration and development of the recommendations, with input from all members. CC, AO’C, and KS were members of a study advisory group who made authorship level contributions to the work. DP led the drafting of the recommendations and this manuscript, with a first draft developed with a core initial writing group including SH-K, CM, AO’C, and CC. All authors contributed to the underpinning research and to subsequent drafts of the recommendations and manuscript and approved the final version. DP acts as guarantor. The corresponding author attests that all listed authors meet authorship criteria and that no others meeting the criteria have been omitted.

Provenance and peer review: Not commissioned; externally peer reviewed.
