# Supplementary material for: Recommendations on recording harms in randomised controlled trials of behaviour change interventions
Source: BMJ. 2024 Oct 2;387:e077418. doi: 10.1136/bmj-2023-077418 (PMC11445694; doi:10.1136/bmj-2023-077418)
Supplement: Supplementary file 1 — Web appendix: Supplementary material [file papd077418.ww.pdf]

Supplementary Material

Contents

1. **Workshop methods** 1

Online workshop participants 1

Online workshop process 1

Online workshop feedback proforma 3

Online workshop feedback summary 3

Full guidance 4

Summarised guidance 5

Implementation and dissemination 5

2. **Categories and mechanisms of harms identified by the systematic scoping review** 6

3. **Supplementary worked example of 1.2 and 1.3** 7

4. **Checklists for identifying and collection harms from behaviour change interventions** 8

References 11

1. Workshop methods

Online workshop participants

Six individuals that took part in the qualitative interviews were invited to take part in the workshops, two of which accepted, three declined and one did not respond. Seventeen additional individuals were identified through reviewing staff listed in the protocols of relevant completed or in progress trials on the National Institute for Health and Social Care Research (NIHR) Funding and Awards. Fifteen of these were invited as recommended by the PSC to ensure we included a range of roles and experience of delivering behaviour change interventions. Nine accepted, four declined, and three did not respond. Following a call to the UK Trial Management Network (TMN), six individuals expressed their interest to take part, all of which were invited to attend. Five attended and one did not respond.

Online workshop process

Two workshops were conducted remotely on Google Meet. The agenda, a feedback proforma (see Figure 1 for example), a more detailed document called 'the full guidance' and a concise version 'the summarised guidance' were shared with the participants one week prior to the workshops. Both workshops followed the following format: Introduction to the workshop, introduction to the RHABIT project and a summary of the findings of the scoping review and qualitative study, and four discussions sessions each focusing on one specific section of the guidance. These discussion sessions focused on the 'Identifying and deciding what harms to record', 'Collecting harms', 'Summarised guidance', and 'Implementation and Dissemination' sections of the guidance. We requested key feedback on whether the attendees felt that there were any gaps in these sections or if they

required any further detail or context, if they felt that they could foresee any challenges in applying the recommendation, and if they had any further comments to add. A pre-meeting was held with the PPI representatives where CM and SHK provided a more in-depth background of the project and its purpose, discussed the guidance in more detail and answered any questions they had prior to the meeting to ensure they both fully understood the topic. The PPI representatives were reimbursed for their time both to review the guidance documents and for their workshop attendance.

Table 1 summarises the attendees for each workshop, their role, and their institution.

Table 1: Workshop Attendees

**Name**	Role	Institution(s)
Workshop 1		
Daphne Babalis	Director of Operations	Imperial CTU, Imperial College London
Lucy Foulkes	Research Fellow	University of Oxford
Rachel Phillips	Senior Lecturer in Medical Statistics and Clinical Trials	Queen Mary University of London
Tom Wainwright	Professor of Orthopaedics and Research Physiotherapist (Chief Investigator)	Bournemouth University and University Hospitals Dorset NHS Foundation Trust
Judith Cohen	CTU Director	Hull Health CTU, University of Hull
Kim Alyousefi-van Dijk	Trial Manager	Anna Freud, London
Ellen Mallender	PPI representative	N/A
Tasleem Aziz	PPI representative	N/A
Workshop 2		
Amy McAndrew	Trial Manager	Exeter CTU, University of Exeter
Chris Bonell	Professor of Public Health and Sociology, NIHR Chief Investigator	London School of Hygiene & Tropical Medicine
Laura Miller	Programme Manager	Pragmatic CTU, Queen Mary University of London
Lucy Clark	Clinical Trial Manager	Norwich CTU, University of East Anglia
Polly Waite	Associate Professor of Clinical Psychology, Principal Investigator	University of Oxford
Alan Montgomery	CTU Director	Nottingham CTU, University of Nottingham

Online workshop feedback proforma

Four topics were discussed in the workshops. These discussion sessions focused on: 'Identifying and deciding what harms to record', 'Collecting harms', 'Summarised guidance', and 'Implementation and Dissemination'. Figure 1 provides an example proforma for the discussion topic: Identifying and deciding what harms to record.

Figure 1: Example proforma (for discussion topic: Identifying and deciding what harms to record)

Items to discuss:

Item	Description
Discussion item<br><br>*E.g. Defining harm*	Description of discussion item<br><br>*E.g., Harms may occur that do not match the GCP definition. We have provided a definition of harm that aims to capture the subjectivity of a harm occurring (potential) and the need for different perspectives (participant + others)*<br><br>*There may be important harms that do not necessarily meet the standard serious criteria i.e., resulting in death, life-threatening episode, hospitalisation etc.*

Suggested feedback matrix:

**Discussion item**	
Gaps/further detail or context required?	
Challenges in applying this recommendation	
Any further comments	

Online workshop feedback summary

The group agreed that having both a full and summarised version of the guidance is very helpful. They felt that the trial manager is likely to read the full document but a summarised version can then

be shared with oversight committees/co-investigators to introduce the area, facilitate discussions, and act as a rationale for the decisions made.

Full guidance

Participants suggested including an executive summary in the full guidance so that readers can quickly understand what it’s for and where it has come from, this is on pages 1 and 2 of the full guidance document.

They felt that the definition of harm we proposed was very helpful, especially by widening this to people beyond the participant. But they queried if we would need to consent those people in order to capture harms to them and their data if so.

Participants also identified potential issues of widening the definition to include those beyond participants and consent issues as they queried if the intervention facilitator or research personnel would then become participants and therefore require CRFs specific to their outcomes and suggested thinking through the practical implications of this. We have added detail about this on page 22.

Some suggested including relevant stakeholder examples, including the people delivering the intervention, e.g. school teachers.

The ‘Identifying harms’ section was well received by the author whose work it was based on, they suggested badging the methods as dark logic modelling as that term does seem to have become increasingly well used.

Participants liked predefined and option for open ended questions but they highlighted that the open ended responses will need to be coded to be able to make sense of them, which will also be important for the statisticians analysing the data. They also highlighted potential issues of unblinding in blinded studies become trickier to manage with open ended questions, so we included this as a consideration on page 21.

One participant raised that it is possible that there will be more opportunity for the intervention arm to report harm than control. They suggested aiming to avoid differential opportunity to report but this can be difficult with many BCI trials as increased contact with researchers through intervention delivery.

Participants felt we need to explain the ‘Subjectivity’ point more.

Workshop participants queried what we would advise if the event was already happening before the trial as in drug trials, an existing event (i.e. one that precedes involvement in the trial) becomes an AE if there is a change in status, i.e. it increases in severity), but they were not sure how easy this would be to establish for the types of harm we're likely to see in BCI but worth considering how differentiate between ongoing (which you probably don’t want to capture) and ongoing but worsening (which you do want to capture). We considered this in section 1.3.1 ‘Practical considerations’ on page 14.

Participants advised to make it clear in the guidance that expected events for BCI trials should be listed that might help (which is what the HRA expect). In a drug trial you'd have an SmPC or IB. We added this expectation in the guidance about listing events in the protocol on page 20.

PPI mentioned that they would be more likely to attribute possible harms to other causes if it hadn't been specifically asked about, so suggesting these direct questions is helpful. They also raised the fact that when they have been a participant in a trial, they have frequently not reported to people they felt were unsympathetic. Participants also raised the point of potential power imbalances between data collectors and participants and should make this a point to consider both at data collection but earlier on too to allow people to feel disclose important information. These points are mentioned on page 22 of the full guidance.

Participants suggested adding a category regarding over-reach e.g. a weight loss intervention causing unhealthy weight loss.

Regarding the 'Collecting harms - Flexibility' section of the guidance, participants highlighted that it is important to think about both subjective and objective attribution – for the latter they suggested thinking about what analysis we you plan undertake and how you might identify differences between arms that indicate signals for potential harm. Note here not suggesting hypothesis testing but more about detecting signals.

Summarised guidance

The workshop participants felt that this would be useful for junior researchers, and suggested creating a video of a PowerPoint presentation to go with this, which could be played to trial teams to introduce the topic, which should include the harms definition and harms from study procedures. They also suggested we include an operational considerations section. This has been added on page 7 of the summarised guidance.

Implementation and dissemination

The group liked the idea of short accompanying videos to help explain the guidance.

They suggested making it clearer that open ended questions are to capture events not captured in predefined list.

Regarding the infographic in the summarised guidance, some of the workshop participants found it a bit difficult to read because of how it's been designed, and suggested some redesign to make grouping and ordering more obvious.

Regarding the checklist as part of the summarised guidance, having an operational considerations column would be helpful to remind users of what they need to do and when.

Summarised and full guidance location following acceptance at the BMJ Research Methods and Reporting

During peer and editorial review at the BMJ Research methods and reporting , the authors decided to use the term recommendations rather than guidance.

A summarised form of the recommendations is available on the project website (https://www.sheffield.ac.uk/ctru/current-trials/rhabit) and includes an infographic.

The full recommendations were adapted from the original full guidance document reviewed at the online workshops. The recommendations are within the manuscript accepted by the BMJ Research Methods and reporting: *Papaioannou D, Hamer-Kiwacz S, Mooney C, Sprange K, Cooper C, O'Cathain A. Recommendations on recording harms in randomised controlled trials of behaviour change interventions. 2024;387:e077418. doi:10.1136/bmj-2023-077418.*

2. Categories and mechanisms of harms identified by the systematic scoping review

Box A: Summary of categories and mechanisms of harms from behaviour change interventions identified in the systematic scoping review

**Categories of harms identified in the systematic scoping review** (1–3)	
**Category**	**Example**
Physical or direct (1,2)	Obesity public health interventions have been shown to increase cigarette smoking and growth failure s in low socio-economic children (4)
Psychosocial or Psychological (1–3)	Stigmatisation, victimization, body dissatisfaction and lowered self-esteem in children following obesity interventions (5,6)
Group and social (1)	Grouping individuals together can allow knowledge exchange for example, antisocial behaviour, drug use (1)
Cultural (2)	Any damage to a population's 'way of life', which includes language, arts and sciences, spirituality, social activity, and interactions (2). For example, smoke free bar policies in California were found to have a negative impact on low SES women living near the bars. Increased smoking on the street may have increased their exposure to second-hand smoke & disruptive noise (7).
Opportunity cost[a] (1,3)	It is hard to identify such harms (1); however, they relate to the potential benefits forgone by committing resources to ineffective or less effective interventions or less serious health problems (1,2).
Environmental (2)	Damage or injury to the circumstances, objects, or conditions by which one is surrounded (2), for example air quality or climate change.
Economic (2)	The roll-out of an intervention where the long-term effects are unknown could result in waste of resources e.g., a vaccination programme (8).
Equity (1)	Improving existing physical activity facilities and building new ones that cater for the local community in deprived neighbourhoods resulted in "inequity drift", that is the new facilities were used more by 'affluent outsiders', not the intended population (9).

**Proposed mechanisms or underlying factors by which harm can occur from behaviour change** (2,3,10–13)
• ***Group interventions:*** may lead to knowledge sharing of harmful behaviours that would not have been known otherwise (e.g., self-harm) or can result in stigmatisation of population subsets (e.g., schoolchildren taken out of the classroom may feel targeted).<br>• ***Feelings of failure or self-efficacy***: Individuals may believe their health is not improving despite taking part in an intervention, which may result in shame, stigma, and guilt and worsening of the intended health behaviour.<br>• **Boomerang/rebound effects**: adoption of a health behaviour often opposite to the health behaviour intended to change (for example, the intention to eat healthily results in more junk food eating).<br>• ***Risk compensation***: improvement of one health behaviour may lead to another behaviour, often unhealthier or negative, to compensate. For example, stopping smoking might result in unhealthy eating patterns.<br>• ***Social norms:*** normalisation of unhealthy behaviours in health communication campaigns, for example nine out of ten people do not eat 5 portions of fruit and veg per day.<br>• ***Ignoring of root causes, lack of stakeholder engagement limited or poor-quality evidence of interventions:*** can contribute to failure to identify underlying factors for a health problem example biological, societal an environmental, poorly designed

[a] Rarely established within a randomised controlled trial, might be established through other research methods for example health economic modelling

3. Supplementary worked example of 1.2 and 1.3

Box B: Identifying anticipated harms and defining harm from a behaviour change intervention- a worked example.

**Behaviour change trial (hypothetical)**
**Population:** Obesity prevention in secondary schoolchildren; cluster randomised controlled trial.<br><br>**Intervention:** School-wide multicomponent nutrition and physical activity behaviour intervention involving a 6-week course delivered in the classroom on healthy eating, cooking workshop, increased daily physical exercise.<br><br>**Comparison:** Usual personal, social, health and economic curriculum.<br><br>**Outcome:** BMI change
**Step 1: Theorise**
One mechanism is identified and **plausible**[a] harms identified:<br>• Feelings of failure in ability to follow intervention may lead to shame, stigma, guilt
**Step 2: Search the literature**
**Literature**: A literature review is identified review (4) which lists potential harms from child obesity prevention interventions:<br>• May result in inappropriate and harmful weight control techniques, e.g., starvation, vomiting, laxative abuse, slimming pill usage, and cigarette smoking to suppress appetite and as a substitute for eating.<br>• Explaining the issues associated with being overweight in prevention programs can make children more conscious of their weight and cause self-perceived lack of athletic ability,

therefore causing them to avoid participation in physical education, sport, and physical activity in general.
**Step 3: Include stakeholder input**
**Stakeholders:**<br>• Schoolteachers raise concerns on overweight children being stigmatised or teased.<br>• Parents are worried about stimulating pre-occupation with weight, unhealthy dieting.<br>• Young people also worry about unhealthy dieting.<br>Stakeholders advise that all instances of stigmatisation and teasing are of **concern** [a]; unhealthy dieting is of great **concern** [a].
**Defining harm: Perspectives[a]**
Child, parent/guardian, person delivering the intervention, schoolteacher.
**Defining harm: Serious or important harms[a]**
Although not meeting the GCP definition of serious, any instances of unhealthy calorie counting or dieting will be reported in real-time.

[a] Key concept in defining harm- see 1.3 in main manuscript

4. Checklists for identifying and collection harms from behaviour change interventions

Box C: Guiding principles to identify anticipated potential harms from behaviour change interventions.

Recognising harm is possible from behaviour change interventions

- Has the trial team discussed the possibility of harms arising from the intervention or research procedures?
- See Table 1 in manuscript for examples that demonstrate behaviour change interventions can cause harm. Further examples available in scoping review (14).

Identifying anticipated harms plausible from the intervention

- Has the trial team considered applicable social and psychological theories to anticipate what could go wrong with the intervention?
- Do the mechanisms and categories of harms in Box A (supplementary material) apply to the trial intervention?
- What literature exists for evidence of harm from similar interventions? (*May be non-RCT evidence*)
- What are stakeholders' views on what could go wrong with an intervention and what are the most important harms to record? (*Involve patient and public representatives*)
- Are there potential harms from the trial research procedures?

Consider if anticipated harms identified are captured by the ICH GCP definition of harm (15)

Three factors to consider for harm from behaviour change interventions:

- Can a plausible link be established with the harm?
- Whose perspectives are important to capture to identify if a harm has occurred?

- *For example, the trial participant, significant other, interventionist or other caregiver?* Consent may be required for individuals other than the trial participants
- Is a harm of concern? Harm perception is subjective; PPI input needed to determine what events might be *of concern*.

For serious or important harms:

- Are there important harms which are not classified as serious as per the GCP definition (15) i.e., resulting in death, hospitalisation, life-threatening episode etc.) but which the trial team need to be aware of in real-time?

Identifying harms inherent in the trial population

- What are the events expected in the trial population which might be considered harmful (irrespective of intervention or research procedures)?

Proportionate recording

- Is the approach to recording harms proportionate?
- Have trial oversight committees agreed the approach to harms recording?
- Factors to consider are:
  - How plausible is the link between the potential harm and the intervention (or research procedure)?
  - How frequently might the harm occur?
  - How risky is it to omit an event recording?

For example, it may be reasonable to exempt recording high frequency harms that occur in a trial population and which have limited evidence to be plausibly caused or worsened by the intervention.

Transparency

- Are anticipated/expected harms from the intervention, population and research procedures documented in the trial protocol?
- Are methods of identifying plausible harms (e.g., literature search) documented?
- Have exemptions of harms from recording, including the rationale for doing so, been documented?

Box D: Checklist to guide data collection of anticipated and unanticipated harms in behaviour change trials

Range of methods to collect harms data

- Data collection needs to incorporate collection of anticipated and unanticipated harms
- Trial primary outcome can capture rebound effects i.e., worsening of the health behaviour intended to change intended.
- Include direct questions on harms identified and decided to record. For example, in a school-based group intervention, ask about being taken out of the classroom.
- Consider including a question such as '*did this worry or concern you*?' to assess whether the event was perceived as harmful.
- Open-ended questions can prompt participants to consider unexpected events caused by the intervention. *For example, did anything about taking part in this study make you feel uncomfortable, upset, or hurt in any way?* Take care not to inadvertently unblind trial team members during analysis of open-ended questions.
- Topic guides in nested qualitative studies can include prompts to explore potential harms.
- Ensure transparency of harms recorded and transparent selection (no cherry-picking (16)).

Monitoring and adapting harms data collection

- Periodic review of harms collected to:
  - Identify where there may be over-recording of harms for example, where large numbers of irrelevant events are found to be recorded during the trial.
  - Consider fee*dback* from qualitative research or intervention facilitators that identifies harms not currently being collected.

Training

- Train trial teams in how the harm is defined in the trial, and the scope of events to record including events exempt from recording is important.
- Train data collectors to be empathetic to encourage reporting from participants.

Consider timepoints for data collection to reduce reporting bias

- Ensure the timepoints for data collection are equal for both trial arms to avoid selective reporting bias.

Attempt attribution assessment wherever possible

- Multi-disciplinary team and oversight committee input may be helpful for attribution, that is deciding on if an event is caused by a trial intervention or research procedure.
- Record harms in both the intervention and control arms to allow comparison on the rate of harms.

References

1. Lorenc T, Oliver K. Adverse effects of public health interventions: A conceptual framework. J Epidemiol Community Health. 2014;68(3):288–90.

2. Allen-Scott L, Hatfield J, McIntyre L. A scoping review of unintended harm associated with public health interventions: Towards a typology and an understanding of underlying factors. Int J Public Health [Internet]. 2014;59(1):3–14. Available from: https://ovidsp.ovid.com/ovidweb.cgi?T=JS&CSC=Y&NEWS=N&PAGE=fulltext&D=psyc11&AN=2014-07681-002

3. Cho H, Salmon CT. Unintended effects of health communication campaigns. J Commun [Internet]. 2007;57(2):293–317. Available from: https://ovidsp.ovid.com/ovidweb.cgi?T=JS&CSC=Y&NEWS=N&PAGE=fulltext&D=psyc5&AN=2007-09372-007

4. O'Dea JA. Prevention of child obesity: "First, do no harm." Vol. 20, Health Education Research. 2005. p. 259–65.

5. Atkinson RL, Nitzke SA. School based programmes on obesity. BMJ [Internet]. 2001;323(7320):1018–9. Available from: http://europepmc.org/abstract/MED/11691747

6. Striegel-Moore RH. The impact of pediatric obesity treatment on eating behavior and psychologic adjustment. J Pediatr. 2001;139(1):13–4.

7. Burgess DJ, Fu SS, van Ryn M. Potential unintended consequences of tobacco-control policies on mothers who smoke: a review of the literature. Am J Prev Med [Internet]. 2009;37(2 Suppl):S151-8. Available from: https://ovidsp.ovid.com/ovidweb.cgi?T=JS&CSC=Y&NEWS=N&PAGE=fulltext&D=med7&AN=19591755

8. Balog JE. The moral justification for a compulsory human papillomavirus vaccination program. Vol. 99, American Journal of Public Health. 2009. p. 616–22.

9. Williams O. Identifying adverse effects of area-based health policy: An ethnographic study of a deprived neighbourhood in England. Health Place [Internet]. 2017;45:85–91. Available from: https://ovidsp.ovid.com/ovidweb.cgi?T=JS&CSC=Y&NEWS=N&PAGE=fulltext&D=med14&AN=28319858

10. Ogden J. Do no harm: Balancing the costs and benefits of patient outcomes in health psychology research and practice. J Health Psychol [Internet]. 2019;24(1):25–37. Available from: https://ovidsp.ovid.com/ovidweb.cgi?T=JS&CSC=Y&NEWS=N&PAGE=fulltext&D=med16&AN=27247091

11. Teachman BA, White BA, Lilienfeld SO. Identifying harmful therapies: Setting the research agenda. Clin Psychol Sci Pract. 2021 Mar;28(1):101–6.

12. Parry GD, Crawford MJ, Duggan C. Iatrogenic harm from psychological therapies--time to move on. Br J Psychiatry [Internet]. 2016;208(3):210–2. Available from: https://ovidsp.ovid.com/ovidweb.cgi?T=JS&CSC=Y&NEWS=N&PAGE=fulltext&D=med13&AN=26932481

13. Mollen S, Ruiter RA, Kok G. Current issues and new directions in Psychology and Health: What are the oughts? The adverse effects of using social norms in health communication. Psychol Health [Internet]. 2010;25(3):265–70. Available from:

https://ovidsp.ovid.com/ovidweb.cgi?T=JS&CSC=Y&NEWS=N&PAGE=fulltext&D=med8&AN=20391219

14. Papaioannou D, Hamer-Kiwacz S, Mooney C, Cooper C, O'Cathain A, Sprange K, et al. Recording harms in randomized controlled trials of behavior change interventions: a scoping review and map of the evidence. J Clin Epidemiol [Internet]. 2024;169:111275. Available from: https://www.sciencedirect.com/science/article/pii/S0895435624000301

15. ICH harmonised guideline. INTEGRATED ADDENDUM TO ICH E6(R1): GUIDELINE FOR GOOD CLINICAL PRACTICE E6(R2). 2016.

16. Mayo-Wilson E, Fusco N, Hong H, Li T, Canner JK, Dickersin K. Opportunities for selective reporting of harms in randomized clinical trials: Selection criteria for non-systematic adverse events. Trials. 2019 Sep;20(1):553.
